# Patient-reported outcomes among patients undergoing total hip replacement in an integrated care system and in a standard care system in Region Stockholm, Sweden

**DOI:** 10.1186/s12913-022-08722-8

**Published:** 2022-11-24

**Authors:** J. Agerholm, F. S. Teni, J. Sundbye, O. Rolfson, K. Burström

**Affiliations:** 1 Aging Research Center, Department of Neurobiology, Care Sciences and Society, Karolinska Institutet, Tomtebodavägen 18A, SE – 171 77 Stockholm, Sweden; 2 Equity and Health Policy Research Group, Department of Global Public Health, Karolinska Institutet, Stockholm, Sweden; 3 Health Outcomes and Economic Evaluation Research Group, Stockholm Centre for Healthcare Ethics, Department of Learning, Informatics, Management and Ethics, Karolinska Institutet, Stockholm, Sweden; 4 Department of Orthopaedics, Institute of Clinical Sciences, Sahlgrenska Academy, University of Gothenburg University, Gothenburg, Sweden; 5 Swedish Arthroplasty Register, Centre of Registers Västra Götaland, Gothenburg, Sweden

**Keywords:** EQ-5D-3L, Hip pain, Integrated care, Patient-reported outcomes, Sweden, Total hip replacement

## Abstract

**Background:**

Coordination, cooperation and efficient use of resources is vital for the health- and social care sector if it is to meet the needs of an aging population. Integrated care is a patient-centred approach to provision of care aiming to improve quality of care and overcome fragmented care through co-productive partnerships and may positively affect quality of care and health outcomes, especially among those in need of highly coordinated care services.

**Aim:**

To compare patient-reported outcomes (PROs) among patients undergoing total hip replacement (THR) in the integrated care system in Norrtälje Municipality and in the standard care system in other municipalities in Region Stockholm, Sweden.

**Methods:**

Swedish Hip Arthroplasty Register PRO data during 2008–2015 were compared 1 year after THR among patients (≥50 years) in integrated care (*n* = 407) and standard care (*n* = 3501) systems using linear (EQ VAS score), logistic (EQ-5D-3L dimensions) and negative binomial (hip pain VAS score) regressions. Analyses were adjusted for the preoperative factors age, sex, BMI, ASA class and type of incision.

**Results:**

1-year postoperatively, patients in the integrated care system did not report their health significantly different from patients receiving standard care. Exceptions: Female patients in integrated care reported less problems with self-care (OR:0.52; 0.29–0.96) and patients above 70 years reported more problems with mobility (OR: 1.37; 1.01–1.87).

**Conclusion:**

No significant differences were found between the two care systems for postoperative PROs. A longer follow-up time and analyses by socioeconomic groups would be valuable.

## Background

In high-income countries, health care systems are challenged by the demographic transition leading to an aging population [1, 2]. Reduced mortality at higher ages has led to more and more people surviving into older ages [1], but at the same time a large proportion of the aging population do not spend all of these additional life years in good health.

In Sweden, it is estimated that 66% of the population above 65 years have comorbidities [2, 3], which leads to an increased need for health and social care services and often from different care providers [4]. Coordination, cooperation and efficient use of existing resources is therefore vital for the health and social care sector to meet the needs of an aging population [4].

Nevertheless, health care services have become more and more specialized during the last decades, leading to an increasingly fragmented system [2]. Coordination between health care providers in Sweden is often reported as lacking which can lead to lower quality of care, decreased patient satisfaction with the care provided, and increased costs due to unnecessary treatments arising from poor communication [3]. Furthermore, additional responsibility is put on the patient and relatives to coordinate the contact between health and social care providers, when coordination between providers are lacking [3].

In this context, integrated care has attained increased interest in both Sweden and elsewhere. Integrated care is a patient-centred approach to provision of care aiming to improve quality of care and overcome fragmented care through co-productive partnerships either within the health care sector or between different operating care organisations [5–8]. Integrated care is believed to have a positive impact on the quality of care, especially for those in need of highly coordinated care services [2, 3, 5, 9]. However, due to the lack of robust evaluations, the impact of integrated care on many quality indicators is still uncertain [10, 11].

### Integrated care in a Swedish setting

In Sweden, there are several ongoing integrated care initiatives, with a varying degree of integration and on different levels in the health and social care system. Norrtälje Municipality in Region Stockholm, has developed and incorporated a fully integrated care system at the organisational level, labelled The Norrtälje Model [12]. The cooperation resulted in a new authority ‘Municipal health care and social care in Norrtälje’ (KSON by Swedish acronym) [12], promoting both horizontal and vertical integration of health and social care in Norrtälje Municipality [13].

KSON owns the health care company ‘Vårdbolaget Tiohundra’ that is responsible for the emergency hospital, primary health care, psychiatric care, childcare, elderly care homes, and home care. The purpose of establishing ‘Vårdbolaget Tiohundra’ is to by-pass usual borders often existing between different care providers to create a smoother access and care-flow for patients [12]. In 2009 and 2010, national choice reforms were introduced giving private providers the right to freely establish publicly funded primary care facilities and home care services [14, 15], which made it possible for multiple providers to establish in Norrtälje Municipality. Hence, ‘Vårdbolaget Tiohundra’ was no longer the only provider of health and social care, however, they are still the main provider.

Well-coordinated care is especially important for vulnerable patients with comorbidities and patients in need of care from several different health and social care services [3]. One such vulnerable group is patients with osteoarthritis. Osteoarthritis is estimated to be the fourth leading cause of disability in 2020 in Sweden [16]. Since a few decades, total hip replacement (THR) has made a significant positive change for patients disabled by hip osteoarthritis [17]. Today, when non-surgical treatment options are not sufficient to control symptoms, the main indication for THR surgery is hip pain and poor general health-related quality of life (HRQoL) [17–19].

A patient undergoing THR will meet professionals from both primary care and specialized care and is in need of both follow-up [20] and early recovery rehabilitation [21] after the surgery in order to get long lasting positive effects of a THR. These patients will therefore benefit from a well-coordinated, patient-centred health and social care system.

Patient-reported outcome measures (PROMs) are used to evaluate aspects of the provided care important to the patient [22, 23] and may also be used to improve quality of care and predict future outcomes in geriatric care [24]. As the main indications for a THR among osteoarthritis patients are hip pain and low HRQoL these patient-reported outcomes (PROs) may provide valuable information to increase knowledge on the impact of standard care as well as integrated care.

The aim of this study was to compare postoperative PROs among patients undergoing THR in the integrated care system in Norrtälje Municipality and in the standard care system in the other municipalities in Region Stockholm.

## Method

This longitudinal register-based study is based on pre- and 1-year postoperative data from patients who have undergone a THR either within an integrated care system (Norrtälje Hospital) or at other publicly owned hospitals operating within a standard care system (Södertälje Hospital; Danderyd Hospital; Karolinska University Hospital in Huddinge; Karolinska University Hospital in Solna; Södersjukhuset).

Norrtälje hospital is placed in Norrtälje Municipality which is the most northern municipality in Region Stockholm. The population in Norrtälje differs to some extend from the total population in the rest of Region Stockholm. There is a higher proportion of people with low income, there are a lower proportion of people born outside Sweden, and the proportion of 80+ year olds are slightly lower [25]. The other hospitals are scattered around Region Stockholm and covers all hospitals in Region Stockholm that provides THR.

The integrated care system in Norrtälje is characterised by having funding responsibilities for both health and social care for the entire population in Norrtälje. It has an explicit focus on health promotion and prevention, and health and social care are integrated on an organisational level to achieve greater benefit for patients. Although the integrated care system covers the entire population in Norrtälje there are some specific goals targeting the needs of older adults. These goals have been described by Back et al. [13], and some are listed below:

- *“To get their health and social problems addressed/solved, and also to satisfy wishes related to health, social care and continuation of a good life in general despite age, chronic conditions and multiple morbidities”*
- *“To experience a higher degree of security in their daily life, continuity and coordinated/comprehensive help from health and social professionals, carers and organisations”*
- *“No older person should be a victim of poor integration, be outside of everyone’s responsibility (In Swedish the idiomatic expression is referred “Nobody should fall between two chairs”).”*
- *“Prioritised prevention work”*
- *“Good and safe medication”*

[13]*.*

The main health and social care provider in Norrtälje is the publicly owned company Tiohundra, which provides primary, secondary, and tertiary care as well as social care. All areas are represented in the company’s management board which facilitates communication and collaboration between the different areas.

In comparison, standard care hospitals provide care in a system where health and social care is divided in two separate systems. Health care is organised and financed within the region, and social care as well as some types of rehabilitation (e.g. rehabilitation provided in the home) are organised and financed by the municipality. Further, primary care is organised separately from hospital care.

Data were retrieved from the Swedish Hip Arthroplasty Register (SHAR) where data on THRs have been collected since the 1970s. Data are based on patients self-reported data from both pre- and post-operative surveys as well as medical data registered by health care staff. Between 2009 and 2018 the completeness rate for the register have been between 97 and 99%. The period for the present study was from January 1st 2008 until December 31st 2015. Patients aged 50 years and above, with primary osteoarthritis who had undergone a unilateral THR procedure during this period in one of the chosen hospitals and who had filled out both pre- and 1-year postoperative questionnaires on their PROMs were included. A total of 3908 patients were eligible for this study.

### Patient-reported outcome measures

The generic instrument EQ-5D-3L was used to measure HRQoL. It consists of the descriptive system where the patient reports his/her own health on five dimensions (mobility, self-care, usual activity, pain/discomfort and anxiety/depression) with three severity levels (no, some and severe problems) and a visual analogue scale (VAS) called the EQ VAS. The EQ VAS reflects the patients’ overall assessment of their own health on a scale ranging from 100 (best imaginable health state) to 0 (worst imaginable health state) [26]. A single-index value of health (1 = full health, 0 = dead) for each of the 243 unique health profiles or health states described by the classification system was obtained by employing the Swedish TTO value set for EQ-5D-3L [27].

Hip pain was measured on a VAS (Pain VAS) ranging from 0 (no pain) to 100 (worst pain imaginable). The patients were asked to rate their hip pain during the past 4 weeks [19].

### Other variables

Other factors, such as age, Body Mass Index (BMI), physical health status, walking ability, type of surgery and interventions preceding the surgery (seeing a physiotherapist or attending arthritis school), may also impact on the measured outcomes and were adjusted for in the analyses.

Patients were divided into the following age groups: 50–59 years, 60–69 years, 70–79 years and above 80 years in the descriptive analyses. When controlling for age in the regression models age was used as a continuous variable.

BMI was categorised into underweight (BMI < 18.5), normal weight (BMI 18.5–24.9), pre-obesity (BMI 25–29.9), obesity class I (BMI 30–34.9), obesity II (BMI ≥35). As there were only one patient being underweight operated on Norrtälje Hospital, we excluded the underweight in the final regression analyses.

All patients were classified according to The American Society of Anaesthesiologists physical status classification (ASA) from class I (healthy) to V (life-threatening) used globally as a standard in preoperative assessments [28]. In this study, no patients met the criteria for ASA class V. ASA class III and IV were combined in the regression analyses as there were too few patients in class IV.

The Charnley classification groups patients into three categories with respect to their walking ability: one hip involved (Charnley class A), both hips involved but no other joints (Charnley class B), and other medical factors contributing to limited walking ability (Charnley class C). The classification system was originally designed for categorization by a professional, however in SHAR it is self-reported by the patient [29].

Several types of incision approaches may be used when performing a THR [30]. In this study, the main part of the incisions performed were a posterior approach or a direct lateral approach. In previous studies, the type of incision approach that is used has been shown to have an impact on PROMs, with a posterior approach leading to slightly better PROMs compared to a direct lateral approach [31]. We therefore use incision type as a control variable in the analyses.

Patient-reported attendance at supported osteoarthritis self-management programme (SOSP) was registered from 2011 [30]. In SOSP the patients attended three theory-based meetings led by a physiotherapist and were offered an individualized exercise-program. Patient-reported information on seeing a physiotherapist or not before surgery was registered in SHAR from 2011 [30].

### Statistical analyses

Data on the percentage of problems reported on the EQ-5D-3L dimensions, EQ VAS score and pain VAS score were analysed pre- and 1-year postoperatively. For the EQ-5D dimensions, Chi-squared test or Fisher’s exact t-test were used. For EQ VAS score and pain VAS score the Mann-Whitney U test was performed. A significance level of < 0.05 was employed.

In order to examine the association between having a THR in an integrated care system and the change in health-related quality of life, multiple linear regression was performed for EQ VAS score and EQ-5D-3L index. Assumptions of normality, linearity and homoscedasticity were examined [32].

The relative odds of reporting some problems or severe problems in each EQ-5D dimension were examined using logistic regression.

The hip pain VAS scale outcome had a high proportion of zeros and a right-skewed distribution. In such circumstances generalised linear models or zero-inflated models have been shown to provide a better fit than standard linear regression models [33]. Although hip pain VAS scores are not counts, they have the same distributional characteristics as count data, as they consist of non-negative integers [33]. We found that the negative binomial regression provided an acceptable fit to the hip pain VAS data. A strength in relation to the zero-inflated models is that the outcomes from the negative binomial regression have been shown to be comparable with those from linear regression [33].

Analyses including the variables attendance at SOSP and visit to physiotherapist was only done on a reduced sample from 2011─2015.

The analyses were conducted using SAS software version 9.4 (SAS Institute Inc., Cary, NC, USA).

## Results

Sociodemographic characteristics and use of physiotherapy for patients in the two systems, integrated care and standard care, are reported in Table 1.

**Table 1:** Sociodemographic characteristics and use of physiopherapy of respondents in Norrtälje Hospital and standard care hospitals, *n* = 3908

	Norrtälje Hospital		Standard care hospitals		
	***n*** = 407		***n*** = 3501		
	%	n	%	n	***P***-value
**Sex**					
Female	57.2	233	58.6	2053	0.590^a^
**Mean age (SD)**	71.7 (8.0)		71.0 (9.2)		0.155^b^
**Age group (years)**					
50–59	7.6	31	12	421	**0.043**^a^
60–69	32.2	131	31.1	1088	
70–79	42	171	37.8	1322	
80+	18.2	74	19.1	670	
**Body mass index, mean (SD)**	27.7 (4.5)		27.1 (4.5)		
**BMI category**					
< 18.5	0.2	1	0.7	26	**0.010**^a^
18.5–24.9	26.3	107	34.1	1195	
25–29.9	43.7	178	40.6	1422	
30–34.9	18.2	74	16.1	564	
> 35	7.9	32	6.2	218	
Missing	3.7	15	2.2	76	
Supported osteoarthritis self-management programme*					
Yes	14.9	35	14.8	313	0.268^a^
No	67.2	158	62.8	1329	
Missing	17.9	42	22.4	474	
Physiotherapist^*^					
Yes	42.1	99	46.7	989	**0.014**^a^
No	40	94	30.9	653	
Missing	17.9	42	22.4	474	

^*^ Collected between 2011 and 2015, Norrtälje hospital *n* = 235, Standard care hospitals *n* = 2116

^a^ Chi-Square test

^b^ Mann-Whitney U test

Baseline clinical information is reported in Table 2. A significantly higher proportion of patients had a THR with a posterior approach in the integrated care system (61%) compared to standard care (46%) (*p* < 0.001). The two groups of patients were comparable regarding ASA and Charnley classifications as well as hip side for operation.

**Table 2:** Clinical information of respondents in Norrtälje Hospital and standard care hospitals, *N* = 3908

	Norrtälje Hospital		Standard care hospitals		
	***n*** = 407		***n*** = 3501		
	%	n	%	n	***P***-value^c^
**ASA**^**a**^					
I	12.5	51	13.3	466	0.080
II	50.4	205	51.7	1809	
III	36.4	148	32.2	1128	
IV	0.5	2	1.3	47	
Missing	0.2	1	1.5	51	
**Charnley class**					
A	51.4	209	42.0	1640	0.085
B	5.4	22	7.9	278	
C	43.2	176	45.2	1583	
**Hip side for surgery**					
Right	59.0	240	57.1	1999	0.470
**Type of incision approach**^**b**^					
Posterior approach	61.4	250	46.4	1625	**< 0.001**
Direct lateral position	37.6	153	49.4	1731	
Other incisions	0.9	4	4.0	141	
Missing	0.0	0	0.1	4	

^a^ The American Society of Anaesthesiologists physical status classification (ASA) system

^b^ Other incisions = Direct lateral supine position (Hardinge), direct lateral trochanteroestoemy, MIS/1- approach (back), MIS/1 approach (front), MIS/2-approach, OCM-approach, Watson-Jones (original)

^c^ Chi-Square test

There was a significantly lower proportion of patients with problems in the mobility dimension pre-operatively (89.9% vs 93.2%, *p* = 0.009) in the integrated care system compared to standard care. One year after surgery there were no significant differences between the two groups in any of the five EQ-5D dimensions. There was no significant difference in the mean EQ VAS score between the two care systems preoperatively or postoperatively (Table 3).

**Table 3:** Problems reported on the EQ-5D-3L dimensions and EQ VAS and pain VAS values, preoperative and 1-year postoperative in Norrtälje hospital and Standard care hospitals, *N* = 3908

	Pre-operative					One-year post-operative				
	Norrtälje hospital		Standard care hospitals			Norrtälje hospital		Standard care hospitals		
	***n*** = 407		***n*** = 3501			***n*** = 407		***n*** = 3501		
Dimension	%	n	%	n	***p-***value	%	N	%	n	***p-***value
**Mobility**										
No problems	10.1	41	6.4	224	**0.009**^a^	50.4	205	53.4	1868	0.289^a^
Some problems	89.9	366	93.2	3262		49.6	202	46.4	1624	
Extreme problems	0	0	0.4	15		0	0	0.3	9	
**Self-care**										
No problems	78.9	321	74.3	2600	0.114^a^	92.1	375	89.5	3132	0.161^a^
Some problems	19.9	81	24.6	860		7.6	31	9.7	339	
Extreme problems	1.2	5	1.2	41		0.2	1	0.9	30	
**Usual activities**										
No problems	38.1	155	39.2	1371	0.915^a^	73.7	300	73.2	2562	0.872^a^
Some problems	52.8	215	51.9	1818		24.3	99	24.4	856	
Extreme problems	9.1	37	8.9	312		2	8	2.4	83	
**Pain/discomfort**										
No problems	1.5	6	1.8	64	0.108^a^	37.1	151	39.5	1383	0.432^a^
Some problems	61.7	251	56.2	1968		56.3	229	55.1	1930	
Extreme problems	36.9	150	42	1469		6.6	27	5.4	188	
**Anxiety/depression**										
No problems	57.5	234	55.1	1930	0.447^a^	73.7	300	73.2	2561	0.803^a^
Some problems	39.8	162	41.1	1440		24.8	101	24.9	872	
Extreme problems	2.7	11	3.7	131		1.5	6	1.9	68	
**EQ VAS**										
Mean (SD)	55.5 (21.2)		56.2 (22.0)		0.811^b^	72.2 (20.4)		73.8 (21.)		0.055^b^
**Hip pain VAS**										
Mean (SD)	62.0 (15.0)		64.5 (16.6)		< 0.001^b^	16.0 (19.1)		14.6 (18.7)		0.106^b^

^a^Chi-square test, ^b^Mann-Whitney U test

The mean hip pain VAS score was significantly lower for patients in integrated care preoperatively (*p* < 0.001). One year post-operatively, there were no significant differences in mean hip pain VAS scores (Table 3).

### Regression analysis on EQ VAS score and EQ-5D-3L index

For the two outcomes EQ VAS scores and EQ-5D-3L index multiple linear regression analyses were conducted (Table 4), with Model 1 controlling for preoperative EQ VAS score or preoperative EQ-5D-3L index, Model 2 additionally controlling for age and sex and Model 3 additionally controlling for BMI, ASA classification and type of incision. No significant differences between integrated and standard care were found in the EQ VAS score and the EQ-5D-3L index, which was also the case for the stratified analyses. There seems to be a tendency for older patients in integrated care to have a more positive outcome compared to older patients in standard care in regards to both EQ VAS score and EQ-5D-3L index, especially among men, however these differences were not significant in any of the models.

**Table 4:** The relative difference in 1-year postoperative EQ VAS score and EQ-5D-3L index for patients operated at Norrtälje Hospital compared to patients operated at standard care hospitals (2008─2015)

	EQ VAS score						EQ-5D-3L index					
	Model 1		Model 2		Model 3		Model 1		Model 2		Model 3	
	Estimate	*p*-value	Estimate	*p*-value	Estimate	*p*-value	Estimate	*p*-value	Estimate	*p*-value	Estimate	*p*-value
All ages	−1.36	0.198	−1.16	0.267	−1.54	0.134	−0.005	0.425	−0.003	0.563	−0.003	0.542
All aged 70+	0.62	0.655	0.53	0.704	−0.27	0.845	−0.003	0.690	−0.003	0.649	−0.006	0.448
All aged 80+	3.76	0.141	3.74	0.143	−0.24	0.861	0.010	0.464	0.010	0.466	−0.006	0.454
Women all ages	−1.82	0.204	−1.77	0.214	−2.19	0.122	− 0.005	0.523	−0.004	0.561	−0.004	0.589
Women aged 70+	−0.58	0.749	−0.68	0.706	−1.47	0.420	−0.007	0.486	−0.007	0.471	−0.007	0.443
Men all ages	−0.74	0.630	−0.24	0.873	−0.65	0.660	−0.004	0.609	−0.001	0.858	−0.003	0.709
Men aged 70+	2.45	0.258	2.39	0.270	1.34	0.532	0.002	0.847	0.002	0.867	−0.005	0.686

Model 1: Controlled for preoperative EQ VAS score or preoperative EQ-5D-3L index

Model 2: Controlled for preoperative EQ VAS score or preoperative EQ-5D-3L index, and age and sex

Model 3: Controlled for preoperative EQ VAS score or preoperative EQ-5D-3L index, and age, sex, BMI, ASA class and type of incision

In the analyses of the reduced sample from 2011─2015, attendance at SOSP was associated with a positive outcome for both EQ VAS score and EQ-5D-3L index, especially among men (for EQ VAS score 4.39; *p* = 0.04). Seeing a physiotherapist was not statistically associated with neither EQ VAS score nor EQ-5D-3L index and also reduced the significance of attendance at SOSP, when added to the model. However, the effect of care system did not change when adding the variables to the model (results not shown).

### Regression analyses on each EQ-5D-3L dimension

Logistic regression was used to calculate the odds of having some or severe problems in each EQ-5D-3L dimension (Table 5). Patients in integrated care aged 70 years or above did have a significantly higher odds of reporting problems in the dimension mobility compared to patients in standard care (OR:1.37; 95% CI: 1.01─1.87), whereas female patients in integrated care had significantly lower odds of reporting any problems in the self-care dimension (OR: 0.52; 95% CI:0.29–0.96).

**Table 5:** The odds ratio (OR) of reporting some or severe problems in each EQ-5D-3L dimension, 1 year postoperatively for patients operated at Norrtälje Hospital compared to patients operated at standard care hospitals (2008─2015)

	Mobility		Self-care		Usual activities		Pain/discomfort		Anxiety/depression	
	OR	95% CI	OR	95% CI	OR	95% CI	OR	95% CI	OR	95% CI
All ages	1.21	0.96–1.51	0.74	0.50–1.11	0.95	0.74–1.22	1.15	0.92–1.44	1.03	0.80–1.34
All aged 70+	**1.34**	1.11–1.62	0.66	0.40–1.09	0.94	0.68–1.29	1.00	0.74–1.34	**1.29**	1.04–1.60
All aged 80+	1.03	0.59–1.79	0.61	0.26–1.40	0.75	0.42–1.35	0.86	0.50–1.48	0.80	0.43–1.48
Women all ages	1.24	0.91–1.68	**0.52**	0.28–0.95	1.07	0.77–1.48	1.19	0.88–1.62	0.96	0.69–1.34
Women aged 70+	1.38	0.92–2.06	0.48	0.23–1.00	1.06	0.70–1.60	0.95	0.64–1.40	0.87	0.57–1.33
Men all ages	1.15	0.82–1.62	1.11	0.64–1.93	0.81	0.54–1.22	1.10	0.79–1.54	1.15	0.77–1.73
Men aged 70+	1.26	0.79–1.99	0.97	0.48–1.98	0.78	0.46–1.32	1.06	0.67–1.68	1.33	0.77–2.29

All models controlled for problems reported on the EQ-5D-3L dimensions preoperatively, age, sex, BMI, ASA class and type of incision

Patients in integrated care aged 70 year or above had significantly higher odds of reporting problems in the anxiety/depression dimension (OR:1.28; 95% CI: 1.04–1.60). In the stratified analyses, women in integrated care had lower OR and men had higher OR for reporting problems in the anxiety/depression dimension. However, none of the stratified analyses were significant.

### Regression analysis on hip pain VAS scores

Negative binomial regression was used to model the hip pain VAS score. In all models, patients in integrated care had slightly higher postoperative scores on hip pain VAS compared to patients in standard care hospitals. None of the results were statistically significant, however the results of the third model were significant on a significance level of 0.1 (1.162, *p* = 0.066) (Table 6). None of the stratified analyses showed any significant effect of integrated care on the hip pain VAS scores.

**Table 6:** The relative difference in the level of 1-year postoperative hip pain VAS score for patients operated at Norrtälje Hospital compared to patients operated at standard care hospitals (2008─2015)

	Hip pain VAS score					
	Model 1		Model 2		Model 3	
	Estimate	*p*-value	Estimate	*p*-value	Estimate	*p*-value
All ages	1.127	0.146	1.116	0.159	1.139	0.103
All aged 70+	1.062	0.582	1.051	0.598	1.094	0.383
All aged 80+	0.896	0.570	0.896	0.554	0.961	0.821
Women all ages	1.105	0.363	1.116	0.290	1.150	0.189
Women aged 70+	0.990	0.919	0.990	0.925	1.020	0.900
Men all ages	1.162	0.234	1.127	0.353	1.116	0.407
Men aged 70+	1.197	0.278	1.174	0.328	1.209	0.249

Model 1: Controlled for preoperative hip pain VAS score

Model 2: Controlled for preoperative hip pain VAS score, age and sex

Model 3: Controlled for preoperative hip pain VAS score, age, sex, BMI, ASA class and type of incision

Neither attending SOSP nor seeing a physiotherapist was associated with any significant change in the hip pain VAS score and the analyses on the reduced sample from 2011─2015 did not differ significantly from the full sample.

### Sensitivity analysis

To account for some of the differences in the socioeconomic composition between Norrtälje Municipality and the comparison area we performed a sensitivity analysis comparing patients in integrated care from Norrtälje Hospital with patients in standard care from Södertälje Hospital. Södertälje Municipality is in the southernmost part of Region Stockholm and more comparable to Norrtälje Municipality in terms of socioeconomic composition. Like Norrtälje Municipality, there is only one hospital. The number of surgeries for THR are almost the same in the two hospitals, however, at Södertälje Hospital they do not perform THR with the posterior approach and we were therefore only able to compare the surgeries from Norrtälje Hospital where they used the direct lateral approach. These analyses did not show any significant difference between the two hospitals.

## Discussion

The findings of this study suggest that patients undergoing THR in integrated care system at the hospital in Norrtälje Municipality did not have a more positive outcome on PROMs compared to patients in standard care hospitals in the other municipalities in Region Stockholm. After surgery, patients in the integrated care system had slightly worse PROMs than patients receiving standard care in many of the outcome measures, however these differences were not statistically significant. The analyses stratified by sex and age did also not show any substantial differences between patients from the two types of care system for most outcomes. Two of the subgroup analyses on the EQ-5D-3L dimensions showed a significant difference between the two groups of patients. Patients aged 70 years or above receiving integrated care reported significantly more problems in the mobility dimension and female patients reported significantly fewer problem in the self-care dimension.

Patients undergoing a THR due to osteoarthritis are in need of early prognosis, care from different health professionals, and early rehabilitation. They may need extra care after surgery, and they often have comorbidities. This group of patients is therefore considered to be a group that could potentially benefit from integrated care as it is provided in Norrtälje Municipality. The findings in this study are therefore somewhat surprising. However, there are some limitations that needs to be considered.

One limitation is that we have no information in this study on the details of the chain of care in the different settings, how THR care was planned and actually implemented. Theoretically, in an integrated care setting, the different care providers involved might be expected to collaborate more closely than in a standard care system. However, this information was not available in our data. Use of pain medication could have impacted on the outcomes. Unfortunately, we have no information on whether the use of pain medication differed between integrated and standard care, which is a limitation with this study.

Socioeconomic position is highly correlated with health. For example, people with lower education often have worse health status than people with higher education [34–36]. They often have poorer compliance to medical treatment [37] and might therefore differ in their ability to follow suggested rehabilitation measures. From previous studies, we know that the proportion of people with low socioeconomic position is higher in Norrtälje Municipality than in the rest of Region Stockholm and this might have affected the results [25]. Data on socioeconomic position was not collected in SHAR; however, we were able to compare Norrtälje Municipality with Södertälje Municipality where the socioeconomic composition is more comparable. We found no differences between the two areas; however, we were only able to compare surgeries using the direct lateral approach as this was the only one used in Södertälje.

Knowledge on the patient’s home municipality could also have added valuable information to this study. It is possible that patients from Norrtälje Municipality could be part of the population receiving the surgery at standard care hospitals, as patients are free to choose providers in other municipalities within Region Stockholm. Although the opposite could also be true, it is more unlikely that patients from municipalities outside Norrtälje would prefer to go to Norrtälje Hospital due to its remote location in the region. Nevertheless, the lack of information on the patients’ home municipality could potentially bias the results if the patients choosing hospitals outside Norrtälje differ significantly from those receiving care at Norrtälje Hospital. It is possible that patients with higher socioeconomic position have better prerequisites for seeking out alternative hospitals for THR and thereby benefit more from the free choice of provider that exists within Region Stockholm. These patients might theoretically also have a better health outcome after surgery.

A strength of this study was the large coverage in the SHAR register giving the possibility to collect data for the entire population in the Region Stockholm. Validation analyses showed 97 to 99% completeness of registrations during the study period [30].

Another strength with the SHAR data is that it gives the possibility to control for many different preoperative characteristics that usually affects the outcomes measured. Comorbidity is one such factor, known to influence surgical outcome [38] and PROMs [34, 39]. Although the Charnley classification cannot be regarded as a proper comorbidity index, previous studies have shown that when the patients preoperative PROMs and Charnley category is known, controlling for further preoperative comorbidity adds little to the prediction of postoperative PROMs [38]. A generic instrument like EQ-5D encompasses different dimensions of health and allow comparisons across diseases. However, disease-specific instruments capture outcomes that might be more closely related to the disease and may be more sensitive to change. Further studies could also include disease-specific instruments.

Although the structure for integrating health and social care was put in place already in 2006, it has been a long process to implement all the different parts of the system that is called the Norrtälje Model today. When the implementation started in 2006, the elderly care services had a financial deficit. This affected the entire integration project and the deficit together with structural changes at the hospital hampered the integration of e.g. primary care [40]. It is therefore uncertain when the integration of the health and social care system in Norrtälje had reached a level where it is reasonable to expect an effect on the type of outcomes investigated in this study.

An evaluation from 2012 showed that only parts of the health and social care sector in Norrtälje Municipality worked in an integrated manner while other parts were still lagging behind [40]. As the time frame of this study was 2008─2015, it is possible that not all patients included were getting their THR in a fully developed integrated care system, which could explain some of the results. In an attempt to account for possible trend changes over the time span of this study, we looked at trends in the relative difference between postoperative and preoperative measures of both EQ VAS scores and hip pain VAS scores over time. Although there were slightly more variations over the years for patients from Norrtälje Hospital, probably due to a lower number of THRs, there were no significant differences in the time trend in the two types of hospital.

It is known that volume of surgeries affects the outcome of surgical, medical, and cardiovascular adverse events after a surgery; the more surgeries a surgeon performs, the fewer adverse events [30, 41]. However, in this study it was not possible to get information on the experience of the surgeons at the different hospitals. If doctors with more experience would be more prone to work in larger hospitals than Norrtälje Hospital, this might influence the results of this study.

Integrated care may be especially important for vulnerable groups, e.g., frail older people, patients with multiple health and psychosocial problems or patients that for different reasons have difficulties navigating in a standard care system. In future studies, socioeconomic position, level of health literacy and frailty could be important aspects to consider when investigating the effect of integrated care on THR. Also, a longer follow-up time could be of interest in order to assess if these differences persist after the initial rehabilitation period.

## Conclusion

This study aimed to increase knowledge on whether and how PROs among patients undergoing a THR in an integrated care system in Norrtälje Municipality differed from PROs among patients in a standard care system. Measures of generic quality of life as well as experienced level of pain 1 year after the operation were evaluated and no significant differences were found between patients undergoing THR in an integrated care system compared to patients undergoing THR in standard care. Further studies with a longer follow-up time could be of interest, in order to assess if these results persist after the initial rehabilitation period. As the effect of integrated care might be more profound for the oldest old, with both health and social care needs, further studies focusing on this group might be of interest as well as subgroup analyses on patients in different socioeconomic position.

## Data Availability

Data can be accessed by applying through the Swedish National Quality Registry for Hip Arthroplasty.

## Abbreviations

ASA: The American Society of Anaesthesiologists physical status classification
BMI: Body mass index
HRQol: health-related quality of life
PRO: Patient-reported outcome
PROM: Patientreported outcome measure
SHAR: Swedish Hip Arthroplasty Register
SOSP: Supported osteoarthritis self-management programme
THR: Total hip replacement
VAS: Visual analogue scale
